# An interpretable machine learning model for MRI-based morphological classification of lumbar disc herniation using functional biomechanical features

**DOI:** 10.3389/fbioe.2026.1856872

**Published:** 2026-10-05

**Authors:** Guanghui Yuan, Can Cui, Yong Xu, Bin Xue

**Affiliations:** 1 School of Economics and Management, Shanghai University of Political Science and Law, Shanghai, China; 2 Department of Traumatology, Ruijin Hospital, Shanghai Jiao Tong University School of Medicine, Shanghai, China

**Keywords:** biomechanical features, interpretability, lumbar disc herniation, machine learning, MRI-based morphological classification, surface electromyography

## Abstract

**Introduction:**

Lumbar disc herniation (LDH) is commonly assessed using magnetic resonance imaging (MRI), although disc morphology does not necessarily correspond to pain intensity, functional impairment, neurological findings, or prognosis. This retrospective single-center exploratory study examined whether functional biomechanical indicators were associated with MRI-based morphological classification of LDH.

**Methods:**

A total of 58 patients were included, comprising 15 patients with disc bulging and 43 with disc protrusion or extrusion. Five predictors—lumbar extension, straight-leg-raising asymmetry, side-flexion asymmetry, gluteus medius asymmetry, and right Jiaji regional surface electromyography root mean square—were used to construct an interpretable L2-regularized logistic regression model. Model performance was evaluated using repeated stratified five-fold cross-validation repeated 10 times, with participant-level out-of-fold probabilities averaged across repetitions. Predictors were selected before cross-validation; therefore, the reported performance may include selection-induced optimism.

**Results:**

The model achieved a receiver operating characteristic area under the curve of 0.705 (95% confidence interval: 0.537–0.851), a precision-recall area under the curve of 0.844, and a Brier score of 0.218. At the exploratory Youden threshold of 0.566, balanced accuracy was 0.714, sensitivity was 0.628, specificity was 0.800, positive predictive value was 0.900, and negative predictive value was 0.429. A prespecified threshold of 0.500 yielded a balanced accuracy of 0.692, sensitivity of 0.651, and specificity of 0.733. Calibration was suboptimal, with an intercept of 0.999 and a slope of 0.752. In a separate exploratory bootstrap analysis, coefficient sign stability ranged from 97.0% to 99.9%, although the intervals remained wide.

**Discussion:**

These findings suggest preliminary associations between selected functional biomechanical indicators and MRI-based disc morphology. Given the small and imbalanced sample, predictor-selection optimism, limited negative predictive performance, suboptimal calibration, and absence of external validation, the model should be regarded as hypothesis-generating rather than as a standalone clinical decision-support tool.

## 1 Introduction

Lumbar disc herniation (LDH) is one of the most common degenerative spinal disorders. It typically manifests as low back pain, radicular symptoms in the lower extremities, and varying degrees of functional impairment. Disease progression can significantly reduce patients’ quality of life and working ability (Ge et al., 2019). According to the Global Burden of Disease study, low back pain and related spinal disorders are still major causes of disability and reduced quality of life worldwide. More and more people are affected, and the condition is occurring at a younger age (Vos et al., 2020). Therefore, objective functional assessment methods may help characterize the relationship between MRI-based morphology and functional manifestations of LDH. Functional tests such as the straight-leg-raising (SLR) test are routinely used in clinical practice to evaluate neural tension and functional limitation in patients with suspected lumbar nerve root involvement.

Magnetic resonance imaging (MRI) remains the primary method for diagnosing and classifying lumbar disc herniation (LDH), as it clearly shows disc structure and nerve root compression. However, growing evidence indicates that the severity of imaging changes does not fully correspond to the level of pain or functional disability (Jensen et al., 1994; Boden et al., 1990). Significant imaging abnormalities can be seen in people without any symptoms, while patients with severe symptoms may show only mild changes on imaging (Stochkendahl et al., 2018). As a result, relying solely on imaging findings may not fully reflect the functional impact of the disease, which limits their value in fully characterizing functional manifestations of the disease. The lumbar anatomical segments relevant to MRI-based morphological assessment are illustrated in Figure 1.

**FIGURE 1:**
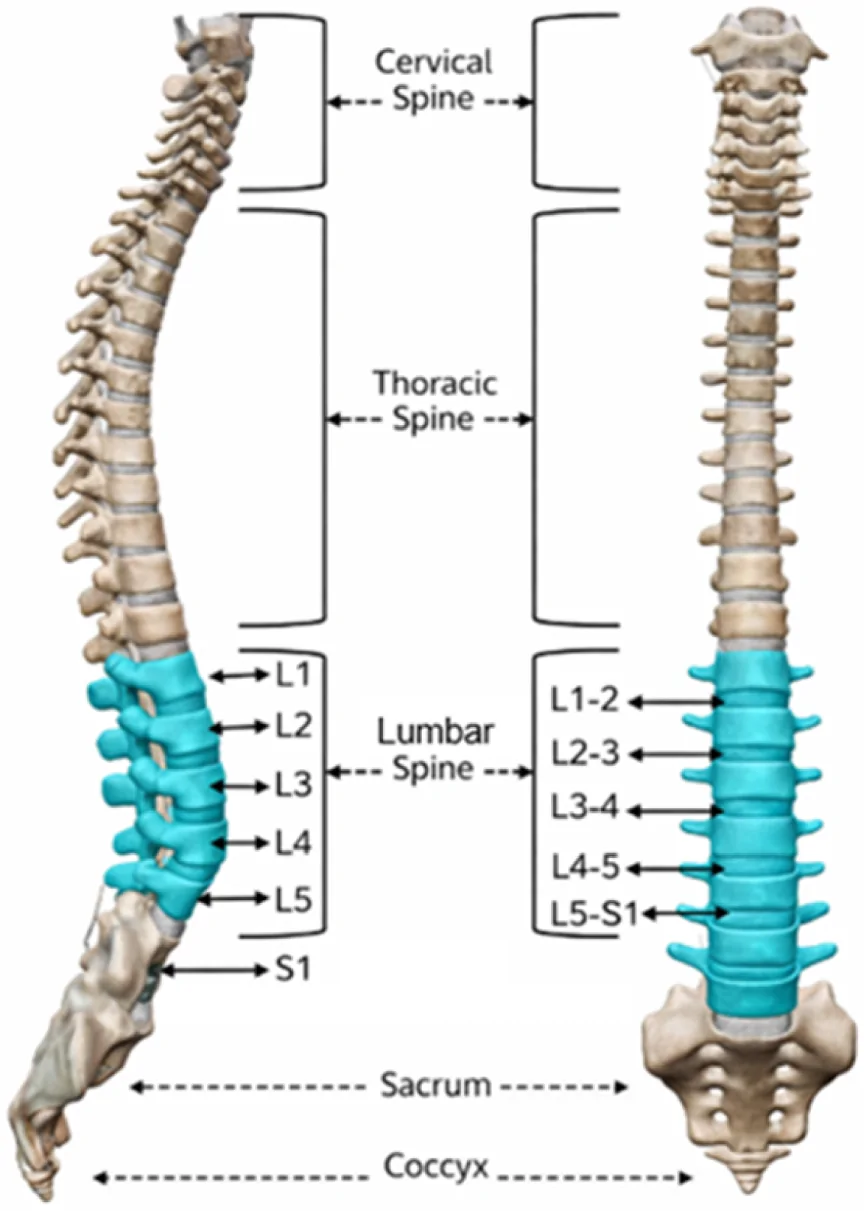
Anatomical overview of lumbar segments involved in MRI-based morphological assessment of lumbar disc herniation.

From a biomechanical viewpoint, lumbar stability relies on the coordinated function of bony structures, ligaments, and muscles. According to Panjabi’s spinal stability theory, dysfunction in any of these systems can reduce stability and lead to pain (Panjabi, 1992). Previous studies have shown that deep trunk muscles, such as the erector spinae and multifidus, play a key role in maintaining dynamic stability of the lumbar spine. Their dysfunction is closely linked to chronic low back pain and the progression of lumbar disc herniation (Hodges and Richardson, 1996). Functional signs of instability including poor postural control, increased body sway, and movement asymmetry have been shown to relate to pain-related functional status and recovery (Ruhe et al., 2011; Ruhe et al., 2012). These findings suggest that functional and kinematic indicators can provide supplementary information beyond imaging evaluation in assessing the impact of the disease.

Traditional Chinese medicine (TCM) classifies lumbar disc herniation (LDH) under the categories of “low back pain” and “bi syndrome.” Its pathogenesis is often attributed to imbalance of the muscles, tendons, and bones (Sun et al., 2014). Wei’s traumatology framework emphasizes the integrated regulation of tendons and bones, highlighting the intrinsic relationship between structural abnormalities and functional dysfunction. (Li, 2015). Clinical treatments based on this theory have been shown to improve pain and functional outcomes (Xue et al., 2023). Functional tests such as the straight-leg-raising (SLR) test are routinely used in clinical practice to evaluate neural tension and functional limitation in patients with suspected lumbar nerve root involvement (Zhang and Zhao, 2014; Deville et al., 2000; Harrison et al., 2004).

In recent years, machine learning methods have been widely used in the analysis of spinal images. Deep learning models can accurately identify and classify lumbar disc herniation automatically based on lumbar MRI scans (Su et al., 2022; Yilihamu et al., 2025; Guo et al., 2025; Xu et al., 2025), multimodal models that combine both imaging and clinical information have also led to further improvements in diagnostic performance (Zhou et al., 2019; Hou et al., 2023). With the emergence of vision transformers and large language models, intelligent assessment of complex spinal disorders has become an active research area (Chu et al., 2025; Dong et al., 2024). Nevertheless, most existing studies primarily focus on imaging data, while the integration of multidimensional functional and biomechanical indicators remains limited. Moreover, under small-sample conditions, complex models may be prone to overfitting, and their lack of interpretability may hinder clinical adoption.

Based on these considerations, this study aimed to develop an interpretable, low-dimensional logistic regression model to explore the association between selected functional biomechanical indicators and MRI-based morphological classification of LDH. The study was designed as a retrospective single-center exploratory analysis rather than a definitive clinical prediction model. We hypothesized that functional range-of-motion and surface electromyography-derived indicators may show preliminary associations with MRI-based disc morphology.

## 2 Materials and methods

### 2.1 Study design and ethical approval

This retrospective single-center study included patients diagnosed with LDH by lumbar MRI at Ruijin Hospital, Shanghai Jiao Tong University School of Medicine, between December 2021 and January 2024. The study protocol was approved by the Ethics Committee of Ruijin Hospital, Shanghai Jiao Tong University School of Medicine (Approval No. 2024-285). Because this study used anonymized retrospective clinical data and involved minimal risk to participants, the requirement for written informed consent was waived by the ethics committee. All clinical data were anonymized before analysis, and the study was conducted in accordance with the Declaration of Helsinki.

### 2.2 Participants and MRI-based morphological outcome definition

Eligible participants met the diagnostic criteria for LDH and were aged between 18 and 60 years. No restrictions were imposed on sex or disease duration. Exclusion criteria included: (1) failure to meet diagnostic criteria for LDH; (2) presence of other lumbar or spinal disorders potentially affecting pain or functional assessment, such as piriformis syndrome, lumbar spinal stenosis, spinal tumors, infections, or previous lumbar surgery; (3) local trauma or skin lesions at the testing sites; (4) pregnancy or severe systemic diseases; and (5) any other condition deemed unsuitable for testing by the investigators.

Lumbar MRI findings were categorized according to disc morphology. Patients with disc bulging were assigned to the bulging group. Patients with disc protrusion or disc extrusion were assigned to the protrusion/extrusion group. This binary outcome was designed to distinguish disc bulging from more focal disc displacement patterns under the limited sample size of the present study.

Importantly, this grouping represented an MRI-based morphological classification rather than a direct measure of clinical disease severity. Disc morphology does not necessarily correspond to pain intensity, disability, neurological deficit, or treatment prognosis. Therefore, the terms “bulging group” and “protrusion/extrusion group” refer primarily to MRI morphology and should not be interpreted as a complete clinical severity grading system.

Radiological classification was performed by a senior radiologist with more than 10 years of clinical experience. Due to the retrospective design and availability of existing clinical records, a second independent MRI reader was not available for the current analysis. Therefore, interobserver reliability assessment using Cohen’s kappa was not performed. This limitation is acknowledged in the Discussion. Patients with concurrent L4–L5 and L5–S1 involvement were retained as a multilevel category. Complete symptomatic side-level correspondence was not consistently available for all patients because of the retrospective design.

The final cohort comprised 58 participants, including 15 in the bulging group and 43 in the protrusion/extrusion group. Given the small and imbalanced sample, the predictor set was restricted and all multivariable analyses were considered exploratory.

### 2.3 Functional and surface electromyography measurements

Surface electromyography (sEMG) signals and functional biomechanical parameters were recorded using a Desktop DTS surface electromyography acquisition system (Noraxon USA Inc., Scottsdale, AZ, USA). The system consisted of eight channels, and sEMG signals were acquired at a sampling frequency of 1500 Hz. Surface electrodes were used for signal acquisition. To ensure data consistency, all measurements were performed using the same device and standardized clinical procedures.

The collected indicators included sEMG-derived muscle activation parameters, regional sEMG measurements, lumbar range-of-motion (ROM) parameters, and neural tension-related functional indicators. Raw sEMG signals were processed using the device-associated preprocessing workflow. The root mean square (RMS) value was extracted as the primary parameter reflecting the magnitude of electromyographic activity. The biomechanical coordinate system and anatomical reference planes used for functional movement measurement are illustrated in Figure 2. The collected functional and sEMG indicators are summarized in Table 1.

**FIGURE 2:**
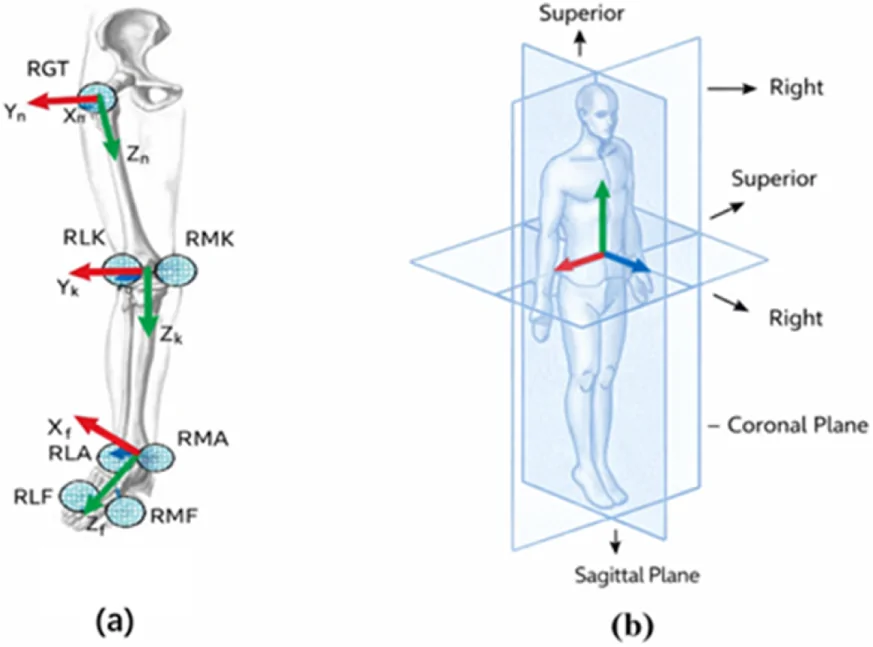
Biomechanical coordinate system and anatomical reference planes used for functional movement measurement. **(a)** Limb feature points and joint coordinate system; **(b)** anatomical planes and movement directions. Anatomical landmark abbreviations shown in the figure indicate representative lower-limb feature points used for coordinate definition.

**TABLE 1:** Raw collected functional and surface electromyography indicators.

Category	Variable	Side	Unit
sEMG	Erector spinae RMS	Left/Right	μV
	Gluteus medius RMS	Left/Right	μV
	Gastrocnemius RMS	Left/Right	μV
Regional sEMG	Interspinous region RMS	—	μV
	Paraspinal Jiaji region RMS	Left/Right	μV
	Juliao region RMS	Left/Right	μV
	Weizhong region RMS	Left/Right	μV
ROM	Flexion	—	​
	Extension	—	degree
	Lateral flexion	Left/Right	degree
	Rotation	Left/Right	degree
Neural tension	Straight leg raise (SLR)	Left/Right	degree

sEMG, surface electromyography; RMS, root mean square; ROM, range of motion; SLR, straight-leg raising.

The anatomical locations of regional sEMG measurements were defined as follows: Jiaji refers to the paraspinal region adjacent to the spinous processes, Juliao refers to the gluteal region, and Weizhong refers to the popliteal fossa region. These standardized anatomical descriptions were provided to improve the interpretability and reproducibility of the measurements for international readers.

Because this study was based on retrospective clinical data, some detailed acquisition parameters, including electrode specifications, interelectrode distance, signal filtering settings, RMS calculation window, skin preparation procedures, normalization methods, assessor blinding procedures, and test–retest reliability assessments, were not fully available. This limitation may affect the reproducibility of the sEMG measurements and is acknowledged in the Discussion. Future prospective studies should adopt standardized sEMG acquisition protocols and report these parameters in detail.

### 2.4 Feature engineering

Feature construction was designed to preserve clinical interpretability and reduce model complexity under small-sample conditions. Because functional alterations associated with LDH may involve bilateral differences in movement control and muscle activation patterns, several derived variables were constructed from left-right measurements.

SLR asymmetry was calculated as the absolute difference between left and right straight-leg-raising angles. Side-flexion asymmetry was calculated as the absolute difference between left and right lateral-flexion angles. Gluteus medius asymmetry was calculated as the absolute difference between left and right gluteus medius sEMG values. These variables were designed to quantify the magnitude of bilateral functional differences rather than directional left-right differences. The definitions of the derived functional features are summarized in Table 2.

**TABLE 2:** Derived functional features.

Feature	Definition
SLR asymmetry	|Left SLR − right SLR|
Side-flexion asymmetry	|Left lateral flexion − right lateral flexion|
Gluteus medius asymmetry	|Left GMed RMS – Right Gmed RMS|

SLR, straight-leg raising; RMS, root mean square. All asymmetry variables were calculated as absolute left-right differences.

### 2.5 Predictor selection

Predictors were selected from the available full dataset before cross-validation based on clinical relevance, biomechanical interpretability, measurement availability, and redundancy. Because selection was not repeated within each training fold, the resampling procedure did not account for selection-induced optimism. Reported performance was therefore considered exploratory cross-validated apparent performance rather than fully unbiased internal validation. Future studies should repeat selection within nested resampling or validate the fixed predictor set in independent cohorts.

### 2.6 Data preprocessing

Continuous predictors were standardized using training-set means and standard deviations within each cross-validation split. Balanced class weights addressed outcome imbalance, and a fixed random seed of 20260722 was applied to all cross-validation and bootstrap procedures. Complete data were available for the analyzed variables; therefore, no imputation was performed.

### 2.7 Model construction

An L2-regularized logistic regression model was used for binary classification. The inverse regularization strength was fixed *a priori* at C=1.0 and was not tuned. The model used the liblinear solver, balanced class weights, and a maximum of 1,000 iterations to estimate the probability of protrusion/extrusion morphology from the selected standardized predictors. The L2-regularized logistic regression model is defined in Equation 1 as follows:

$$\text{logit}\left(P\right)=\ln\left(\frac{P}{1-P}\right)=\beta_{0}+\sum_{i=1}^{k}\beta_{i}X_{i}$$ (1)

where P denotes the probability of protrusion/extrusion morphology, $X_{i}$ represents the selected predictors, and $\beta_{i}$ represents coefficients estimated under L2 regularization.

### 2.8 Performance evaluation metrics

Performance was evaluated using ROC-AUC, PR-AUC, Brier score, calibration measures, and threshold-dependent metrics, including balanced accuracy, sensitivity, specificity, positive and negative predictive values, F1-score, and MCC. Recall, precision, F1-score, and the Youden index were calculated according to Equations 2–5, respectively.

$$\text{Recall}=\frac{TP}{TP+FN}$$ (2)

$$\text{Precision}=\frac{TP}{TP+FP}$$ (3)

$$F1-\text{score}=\frac{2\times Recall\times Precision}{Recall+Precision}$$ (4)

J=Sensitivity+Specificity−1 (5)

Repeated stratified five-fold cross-validation was performed 10 times. Each participant received 10 out-of-fold predicted probabilities, which were averaged to obtain one participant-level prediction. All discrimination, calibration, and threshold-dependent metrics were calculated from these averaged predictions.

The ROC-AUC 95% confidence interval was estimated using 2,000 participant-level nonparametric bootstrap resamples. In each iteration, 58 participants were sampled with replacement from the participant-level averaged out-of-fold predictions, and the ROC-AUC was recalculated. The 2.5th and 97.5th percentiles of the bootstrap distribution were used as the confidence limits.

The exploratory Youden threshold was derived from the participant-level averaged out-of-fold predicted probabilities by maximizing sensitivity plus specificity minus one. These averaged probabilities were dichotomized using the selected threshold to generate one predicted classification for each of the 58 participants and the corresponding confusion matrix. Because the threshold was selected and evaluated using the same averaged out-of-fold predictions, the resulting threshold-dependent metrics may be optimistic and were interpreted exploratorily. A prespecified threshold of 0.50 was additionally evaluated as a sensitivity analysis.

Calibration intercept and slope were estimated by fitting a logistic recalibration model with the observed binary outcome as the dependent variable and the logit-transformed participant-level averaged out-of-fold predicted probability as the independent variable. Predicted probabilities were truncated to the interval from $10^{-6}$ to $1-10^{-6}$ before logit transformation. The intercept and slope were estimated simultaneously, with ideal values of 0 and 1, respectively.

For the calibration plot, participants were divided into five quantile-based groups. Observed proportions were displayed with Wilson 95% confidence intervals. The confidence band for the logistic recalibration curve was estimated using 2,000 participant-level bootstrap resamples conditional on the averaged out-of-fold predictions; the full model-development and predictor-selection procedures were not repeated within this bootstrap analysis.

### 2.9 Exploratory multivariable association and coefficient stability analysis

A separate unpenalized logistic regression model was fitted to explore the direction and approximate magnitude of associations between the five standardized predictors and MRI morphology. This model was distinct from the L2-regularized model used for cross-validated prediction. Coefficient stability was assessed using 1,000 participant-level bootstrap samples. For each predictor, the bootstrap median, percentile-based 95% interval, and sign stability were calculated; 997 samples yielded valid converged estimates. The analysis was conditional on the fixed predictor set and did not incorporate predictor-selection uncertainty. Analyses used Python 3.13.5, scikit-learn 1.8.0, and statsmodels 0.14.6.

## 3 Results

### 3.1 Baseline clinical and demographic characteristics

A total of 58 patients were included in the analysis, including 15 patients in the bulging group and 43 patients in the protrusion/extrusion group. Baseline clinical and demographic characteristics are summarized in Table 3. No statistically significant differences were observed between the two groups in age, BMI, VAS score, ODI score, symptom duration, sex distribution, disc level, or symptom/disc side. These comparisons were descriptive and should be interpreted cautiously because of the limited sample size and imbalance between groups.

**TABLE 3:** Baseline clinical and demographic characteristics of the study population.

Variable	Bulging group (n = 15)	Protrusion/extrusion group (n = 43)	P-value
Age, years	40.40 ± 5.85	37.77 ± 8.28	0.206
BMI, kg/m^2^	25.33 ± 2.98	25.31 ± 2.58	0.817
VAS score	6.73 ± 0.46	6.91 ± 0.75	0.488
ODI score	32.40 ± 2.56	31.70 ± 2.11	0.480
Symptom duration, months	3.20 ± 1.82	3.23 ± 1.82	0.957
Female	9/15 (60.0%)	20/43 (46.5%)	0.550
Male	6/15 (40.0%)	23/43 (53.5%)	-
L4-5	9/15 (60.0%)	19/43 (44.2%)	0.262
L4-S1	0/15 (0.0%)	6/43 (14.0%)	-
L5-S1	6/15 (40.0%)	18/43 (41.9%)	-
Left side	7/15 (46.7%)	18/43 (41.9%)	0.770
Right side	8/15 (53.3%)	25/43 (58.1%)	-

Continuous variables are presented as mean ± standard deviation. Categorical variables are presented as n/N (%). VAS, visual analogue scale; ODI, Oswestry Disability Index; BMI, body mass index. L4–S1 indicates concurrent involvement of the L4–L5 and L5–S1 intervertebral disc levels.

### 3.2 Comparison of selected functional predictors

The comparison of selected functional predictors between MRI-based morphological groups is shown in Table 4. In univariate comparisons, none of the selected predictors reached statistical significance. However, several variables showed small-to-moderate effect sizes. These findings were considered together with prior clinical relevance, biomechanical interpretability, and measurement availability during exploratory model construction.

**TABLE 4:** Comparison of selected functional predictors between MRI-based morphological groups.

Predictor	Bulging group (n = 15)	Protrusion/extrusion group (n = 43)	P-value	Effect size (r)
Lumbar extension	10.67 ± 4.17	13.26 ± 6.35	0.094	0.28
SLR asymmetry	24.33 ± 11.32	25.58 ± 14.23	0.663	0.06
Side-flexion asymmetry	7.33 ± 4.95	5.93 ± 4.26	0.296	0.14
Gluteus medius asymmetry	40.74 ± 18.99	33.53 ± 25.26	0.279	0.16
Right Jiaji regional sEMG RMS	2.43 ± 0.78	2.20 ± 0.73	0.301	0.15

Values are presented as mean ± standard deviation. SLR, straight-leg raising; sEMG, surface electromyography; RMS, root mean square.

### 3.3 Cross-validated model performance

Based on participant-level averaged out-of-fold predictions, the model achieved a ROC-AUC of 0.705 (95% CI: 0.537–0.851), a PR-AUC of 0.844, and a Brier score of 0.218 (Figure 3; Table 5). The majority-class baseline accuracy was 0.741, indicating that accuracy alone was not informative under the observed class imbalance.

**FIGURE 3:**
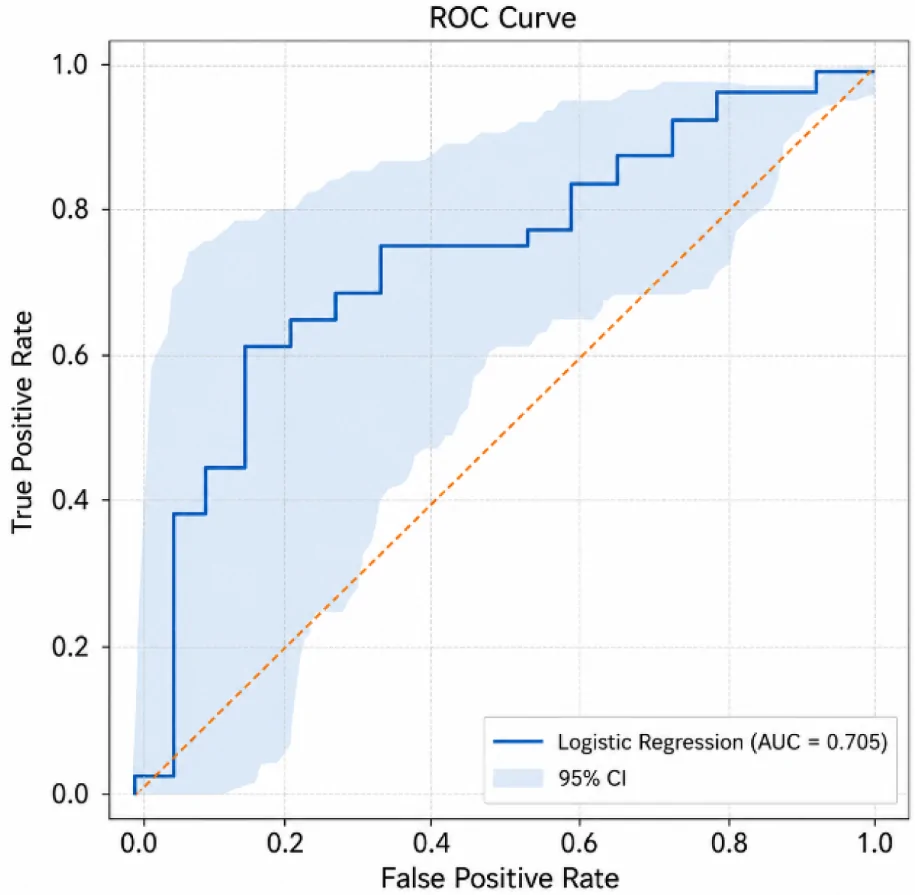
Receiver operating characteristic curve based on participant-level averaged out-of-fold predictions from repeated stratified five-fold cross-validation. ROC-AUC was 0.705 (95% CI: 0.537–0.851), with the confidence interval estimated using 2,000 participant-level bootstrap resamples.

**TABLE 5:** Cross-validated discrimination, calibration, and threshold-dependent performance.

Metric	Value
Panel A. Threshold-independent performance	
ROC-AUC	0.705
ROC-AUC 95% CI	0.537–0.851
PR-AUC/Average precision	0.844
Brier score	0.218
Calibration intercept	0.999
Calibration slope	0.752

Metric	Exploratory youden threshold (0.566)	Prespecified threshold (0.500)
Panel B. Threshold-dependent performance		
Accuracy	0.672	0.672
Balanced accuracy	0.714	0.692
Sensitivity	0.628	0.651
Specificity	0.800	0.733
PPV/Precision	0.900	0.875
NPV	0.429	0.423
F1-score	0.740	0.747
Matthews correlation coefficient	0.375	0.339

Metrics were calculated from participant-level averaged out-of-fold predictions. The exploratory Youden threshold was selected and evaluated using the same predictions and may therefore yield optimistic threshold-dependent estimates. A prespecified threshold of 0.500 was evaluated as a sensitivity analysis. ROC-AUC, receiver operating characteristic area under the curve; PR-AUC, precision-recall area under the curve; PPV, positive predictive value; NPV, negative predictive value; MCC, Matthews correlation coefficient; CI, confidence interval.

At the exploratory Youden threshold of 0.566, balanced accuracy, sensitivity, and specificity were 0.714, 0.628, and 0.800, respectively. At the prespecified threshold of 0.500, the corresponding values were 0.692, 0.651, and 0.733. Complete threshold-dependent results are reported in Table 5. Estimates at the Youden threshold may be optimistic because the same predictions were used for threshold selection and evaluation. The corresponding confusion matrix at the exploratory Youden threshold is shown in Figure 4.

**FIGURE 4:**
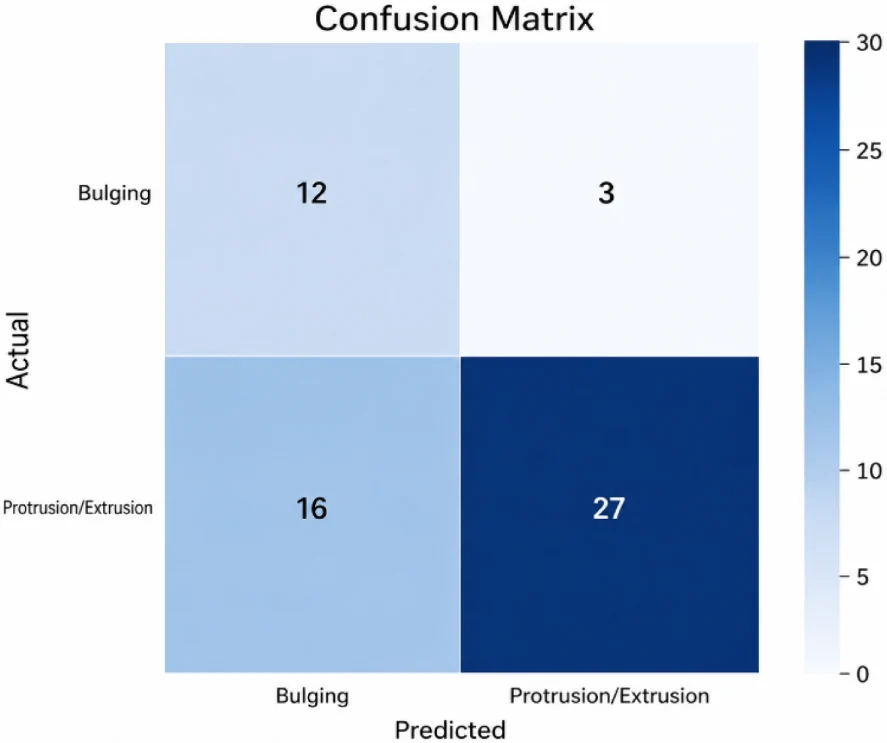
Confusion matrix at the exploratory Youden threshold of 0.566. Rows indicate observed MRI-based morphological groups and columns indicate predicted groups. Because the threshold was selected and evaluated using the same averaged out-of-fold predictions, the matrix should be interpreted exploratorily.

### 3.4 Calibration analysis

Calibration intercept and slope were 0.999 and 0.752, respectively, indicating departure from ideal calibration. Observed protrusion/extrusion proportions were generally higher than mean predicted probabilities across the five quantile-based bins (Figure 5). Calibration was therefore considered suboptimal and interpreted cautiously given the small sample and balanced class weighting.

**FIGURE 5:**
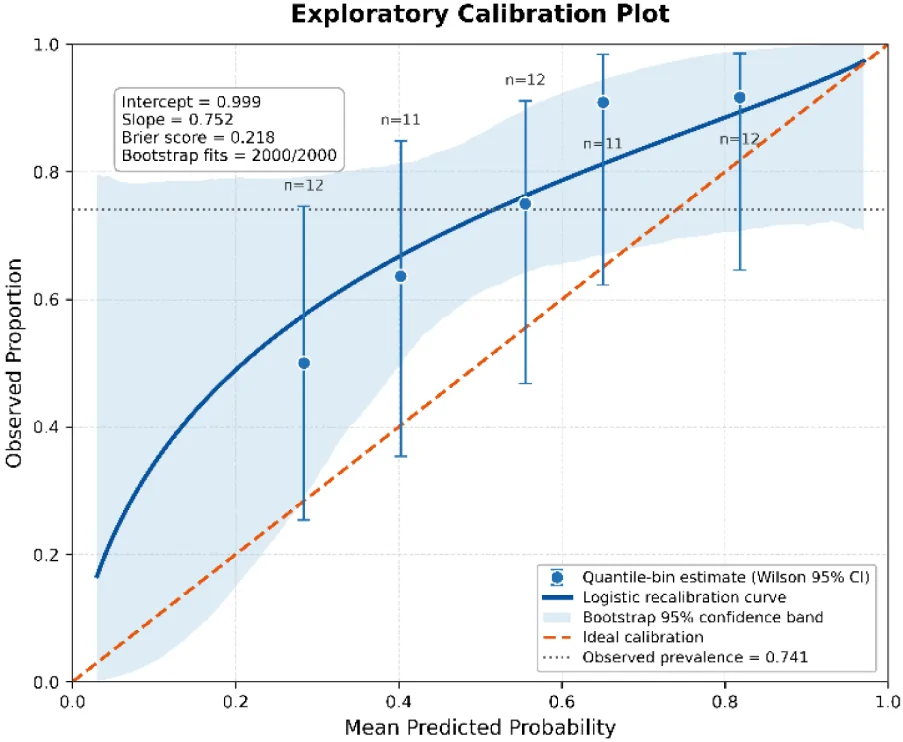
Exploratory calibration analysis based on participant-level averaged out-of-fold predicted probabilities.

Points represent the observed proportions of protrusion/extrusion morphology within five quantile-based groups, with vertical error bars indicating Wilson 95% confidence intervals; the sample size of each group is shown adjacent to the corresponding point. The solid blue curve represents the logistic recalibration function, and the shaded area represents the participant-level bootstrap 95% confidence band based on 2,000 resamples. The dashed diagonal line indicates ideal calibration, whereas the dotted horizontal line indicates the observed prevalence of protrusion/extrusion morphology (0.741). The calibration intercept was 0.999, the calibration slope was 0.752, and the Brier score was 0.218. The bootstrap confidence band was conditional on the participant-level averaged out-of-fold predictions and did not incorporate uncertainty arising from predictor selection or complete model refitting.

### 3.5 Exploratory multivariable association and coefficient stability analysis

Of the 1,000 bootstrap samples, 997 yielded valid coefficient estimates. Sign stability ranged from 97.0% to 99.9%, but the bootstrap intervals were wide, indicating uncertainty in coefficient magnitude. The interval for right Jiaji regional sEMG RMS included zero. These results describe association stability conditional on the fixed predictor set and should not be interpreted as reliable independent effects.

### 3.6 Collinearity assessment

No evidence of severe multicollinearity was observed among the selected predictors, with all VIF values below 5. The VIF values were 1.237 for lumbar extension, 2.150 for SLR asymmetry, 1.909 for side-flexion asymmetry, 1.370 for gluteus medius asymmetry, and 1.292 for right Jiaji regional sEMG RMS. The VIF values for all selected predictors are summarized in Table 6. Moderate correlations were observed among some functional asymmetry variables, especially between SLR asymmetry and side-flexion asymmetry. The correlation assessment and VIF results were used to evaluate potential redundancy among predictors.

**TABLE 6:** Variance inflation factor assessment of selected predictors.

Predictor	VIF
Lumbar extension	1.237
SLR asymmetry	2.150
Side-flexion asymmetry	1.909
Gluteus medius asymmetry	1.370
Right Jiaji regional sEMG RMS	1.292

VIF, variance inflation factor; VIF values below 5 were considered to indicate no evidence of severe multicollinearity.

The VIF analysis was used only to assess multicollinearity and was not considered evidence of coefficient stability. Coefficient stability was evaluated separately using the participant-level bootstrap analysis reported in Section 3.5 and Table 7.

**TABLE 7:** Bootstrap assessment of standardized coefficient stability in the exploratory unpenalized multivariable logistic regression analysis.

Predictor	Full-sample standardized beta	Bootstrap median beta	Bootstrap 95% interval	Sign stability, %
Lumbar extension	0.901	1.044	0.067 to 3.704	98.4
SLR asymmetry	1.535	1.771	0.424 to 5.735	99.9
Side-flexion asymmetry	−1.543	−1.780	−5.683 to −0.526	99.8
Gluteus medius asymmetry	−1.252	−1.425	−4.265 to −0.385	99.7
Right Jiaji regional sEMG RMS	−0.788	−0.917	−3.318 to 0.039	97.0

Coefficients were estimated after z-score standardization of the five predictors. Bootstrap results were obtained using 1,000 participant-level nonparametric bootstrap samples, of which 997 produced valid converged estimates. The bootstrap interval represents the 2.5th and 97.5th percentiles of the valid bootstrap coefficient distribution. Sign stability represents the percentage of valid bootstrap estimates having the same sign as the bootstrap median. The analysis was conditional on the fixed predictor set selected using the available full dataset and did not account for predictor-selection uncertainty. These estimates should be interpreted as exploratory associations and not as reliable independent or causal effects. SLR, straight-leg raising; sEMG, surface electromyography; RMS, root mean square.

## 4 Discussion

This retrospective single-center exploratory study investigated whether selected functional biomechanical indicators were associated with MRI-based morphological classification of LDH. The main finding was that an interpretable logistic regression model using five functional predictors showed moderate discrimination between patients classified into the bulging group and those classified into the protrusion/extrusion group. However, the model also showed limited negative predictive performance and uncertainty in probability calibration. Therefore, the findings should be interpreted as preliminary and hypothesis-generating rather than as evidence for a clinically deployable prediction model.

Although the ROC-AUC and balanced accuracy suggested moderate discrimination, the high naive baseline accuracy reflected the predominance of the protrusion/extrusion group in this dataset. The relatively high PPV was partly influenced by the prevalence of the positive class, whereas the low NPV indicated limited ability of the model to exclude protrusion/extrusion morphology. Probability calibration was suboptimal and should be distinguished from model discrimination. Under the conventional logistic recalibration definition, the calibration intercept of 0.999 indicated calibration-in-the-large error, whereas the calibration slope of 0.752 suggested that the predicted probabilities may have been overly dispersed. The calibration plot also showed that the observed proportion of protrusion/extrusion morphology was generally higher than the corresponding mean predicted probability across the quantile-based groups. These findings suggest that the model tended to underestimate the absolute probability of protrusion/extrusion morphology.

The use of balanced class weights may have contributed to this discrepancy because class weighting was introduced to improve classification under imbalance rather than to preserve probability calibration relative to the observed prevalence. Therefore, the predicted probabilities should not be interpreted as absolute clinical risk estimates, and formal recalibration would need to be evaluated in a larger independent cohort before any clinical application. These findings indicate that the model should not be used as a standalone clinical decision-support tool.

The outcome definition is essential for interpreting the findings. Disc bulging, protrusion, and extrusion represent MRI-based morphological patterns and should not be considered direct equivalents of clinical disease severity. Pain intensity, disability, neurological findings, symptom duration, and treatment response may vary substantially among individuals with similar imaging findings. Therefore, this study explored associations between functional biomechanical indicators and MRI-based disc morphology rather than attempting to classify complete clinical severity.

The exploratory multivariable association analysis should be distinguished from the regularized prediction model. Conventional inferential P-values and Wald confidence intervals were removed because the five-predictor unpenalized model was fitted in a dataset containing only 15 participants in the smaller morphological group. Participant-level bootstrap analysis showed that coefficient directions were retained in most valid resamples, but the bootstrap intervals were wide, indicating substantial uncertainty in coefficient magnitude.

These findings suggest that the observed directions of association may be reasonably consistent conditional on the selected five-predictor set, but they do not establish that the variables are reliable independent predictors. The bootstrap analysis also remained conditional on predictors selected using the available full dataset and therefore did not incorporate predictor-selection uncertainty. The coefficients should consequently be interpreted as hypothesis-generating associations rather than stable inferential estimates or causal effects.

Among the selected predictors, SLR asymmetry may reflect bilateral differences in functional responses during neural tension testing. Side-flexion asymmetry may reflect differences in trunk movement patterns and neuromuscular control. These observations should not be interpreted as direct causal mechanisms but suggest potential functional alterations associated with MRI-based morphological differences.

Lumbar extension showed a positive association with the protrusion/extrusion morphological group in the exploratory multivariable model. This finding should be interpreted cautiously because it may reflect small-sample instability, predictor correlations, compensatory movement patterns, or model uncertainty rather than a direct causal relationship.

The SLR test was selected because it is routinely applied in clinical assessment and was consistently available in the retrospective dataset. However, the SLR test mainly reflects neural tension-related functional responses and may not fully capture disc-related mechanical loading. Additional load-based assessments, such as the compression load test, may provide complementary information regarding disc involvement and mechanical sensitivity. Because the compression load test was not routinely collected in the current retrospective cohort, it could not be included in the present model. Future prospective studies should incorporate compression-based and other load-based biomechanical tests to provide a more comprehensive functional evaluation of LDH.

Several limitations should be acknowledged. This study was limited by its retrospective single-center design, small sample size, class imbalance, potential predictor selection optimism, single-reader MRI classification, incomplete symptomatic side-level information, unavailable detailed sEMG acquisition parameters, limited calibration, and lack of external validation. Therefore, the clinical applicability of the model remains uncertain. In addition, because predictor selection was performed using the available full dataset before cross-validation, the reported model performance may be affected by selection-induced optimism. Therefore, the discrimination and classification metrics should be interpreted as exploratory and potentially optimistic estimates rather than definitive internally validated performance. The original screening log was unavailable, preventing reconstruction of the exact number screened and excluded before formation of the analytic cohort.

Despite these limitations, this study provides preliminary evidence that selected functional biomechanical indicators may be associated with MRI-based disc morphology. The use of a low-dimensional and interpretable logistic regression model may be appropriate for exploratory analysis under small-sample conditions. Future prospective multicenter studies should validate these findings, standardize measurement protocols, evaluate MRI classification reliability, assess calibration, and determine whether functional indicators provide additional information beyond conventional clinical and imaging variables.

## 5 Conclusion

In this retrospective single-center exploratory study, selected functional biomechanical indicators showed preliminary associations with MRI-based morphological classification of LDH. The interpretable logistic regression model demonstrated moderate discrimination, but negative predictive performance and probability calibration remained limited. Given the small sample size, class imbalance, retrospective design, and lack of external validation, these findings should be considered preliminary and hypothesis-generating. Larger prospective multicenter studies are required to validate the stability, calibration, and clinical relevance of these functional indicators.

## Data Availability

The data used in this study are not publicly available due to privacy and ethical restrictions, but are available from the corresponding author upon reasonable request.
